# Investigation of Angiogenic Potential in CD146-Positive Stem Cells Derived from Human Exfoliated Deciduous Teeth

**DOI:** 10.3390/ijms26030974

**Published:** 2025-01-24

**Authors:** Kodai Rikitake, Ryo Kunimatsu, Yuki Yoshimi, Kotaro Tanimoto

**Affiliations:** 1 Department of Orthodontics, Division of Oral Health and Development, Hiroshima University Hospital, 1-2-3 Kasumi, Minami-ku, Hiroshima 734-8553, Japan; 2 Department of Orthodontics and Craniofacial Developmental Biology, Graduate School of Biomedical and Health Sciences, Hiroshima University, 1-2-3 Kasumi, Minami-ku, Hiroshima 734-8553, Japan

**Keywords:** stem cells from human exfoliated deciduous teeth, SHED, angiogenesis, CD146, VEGF, bone regeneration

## Abstract

This study aimed to evaluate the effects of CD146, a surface antigen of mesenchymal stem cells from human exfoliated deciduous teeth (SHEDs), on angiogenic potential. SHEDs were isolated from patients’ deciduous teeth and sorted into CD146-positive (CD146 + SHED) and CD146-negative (CD146 − SHED) populations. Three groups—non-sorted SHED, CD146 + SHED, and CD146 − SHED—were compared. Angiogenic potential was assessed by co-culturing each group with human umbilical vein endothelial cells (HUVECs) and evaluating lumen formation using an endothelial tube formation assay. The gene and protein expression levels of angiogenic markers, including vascular endothelial growth factor (VEGF), VEGF receptor 2 (VEGFR2), cluster of differentiation 31 (CD31), and basic fibroblast growth factor (bFGF), were analyzed using a real-time polymerase chain reaction and enzyme-linked immunosorbent assay. The tube formation assay revealed significantly enhanced angiogenic potential in CD146 + SHED and non-sorted SHED compared to CD146 − SHED. The gene and protein expression levels of VEGF, VEGFR2, CD31, and bFGF were significantly upregulated in CD146 + SHED and non-sorted SHED, highlighting superior angiogenic capabilities in CD146 + SHED. CD146 + SHED demonstrated enhanced angiogenic potential compared to CD146 − SHED, supporting their use in regenerative therapies targeting angiogenesis.

## 1. Introduction

The transplantation of mesenchymal stem cells (MSCs) has gained significant attention in regenerative therapy due to MSCs’ high proliferative potential capacity and ability to differentiate into various cell types, including bone cells, chondrocytes, and adipocytes, making them suitable for treating diverse tissue defects [1,2]. MSCs have been studied extensively for their potential in regenerative therapy targeting various tissue defects [3,4,5]. Therefore, we focused on mesenchymal stem cells derived from human exfoliated deciduous teeth (SHEDs) as a less invasive cell source for patients. SHEDs have high proliferative potential and can differentiate into bone, adipose, cartilage, and nerve cells [6,7]. Previous research demonstrated that SHEDs possess a bone regenerative potential comparable to that of bone marrow-derived mesenchymal stem cells (BMSCs) and human permanent dental pulp stem cells (DPSCs) [8,9]. The surface antigens of MSCs have also attracted attention due to their roles as receptors for growth factors and co-receptors for cell membrane receptors, contributing to various regenerative effects, such as promoting angiogenesis and bone differentiation [10]. MSCs isolated from tissues form heterogeneous populations expressing various surface antigens. Cell sorting enables the isolation of cells expressing specific surface antigens, facilitating the study of their influence on cellular properties [11]. One surface antigen, CD146, is expressed on the plasma membrane of vascular endothelial cells, vascular pericytes, and MSCs and functions as an important cell adhesion molecule in angiogenesis [12,13]. It has been shown that CD146 is not only an adhesion molecule but also a cell surface receptor for a variety of ligands, including several growth factors and extracellular matrices [13]. CD146 is actively involved in many physiological and pathological processes in cells through its bidirectional interactions with ligands [13]. CD146 is a highly glycosylated type I transmembrane protein and belongs to the immunoglobulin superfamily [13]. In 2012, other laboratories showed that CD146 binds to vascular endothelial growth factor receptor 2 (VEGFR2), a co-receptor required for activation by vascular endothelial growth factor A (VEGF-A) [13].

In our previous study, a heterogeneous SHED population, CD146-positive SHED (CD146+) group, and CD146-negative SHED (CD146−) group were isolated by cell sorting and transplanted into a skull defect model in immunocompromised mice. Subsequently, bone regeneration was compared among the three groups using computed tomography (CT) imaging and tissue staining [14]. Additional in vitro experiments, including real-time polymerase chain reaction (PCR), alkaline phosphatase (ALP) staining, and Alizarin red staining, suggested that CD146+ cells exhibited superior bone differentiation ability compared to the other groups [15]. Based on previous reports that CD146 is associated with angiogenesis and the results of our previous studies, we hypothesized that the predominance of bone regeneration in CD146 + SHED may be due to the predominance of angiogenesis. However, the angiogenic potential of CD146 in SHEDs remains unexplored. This study aimed to investigate the angiogenic potential of CD146 in SHEDs by comparing a heterogeneous SHED cell population without cell sorting, CD146 + SHED, and CD146 − SHED.

## 2. Results

### 2.1. Tube Forming Assay

**Figure 1:**
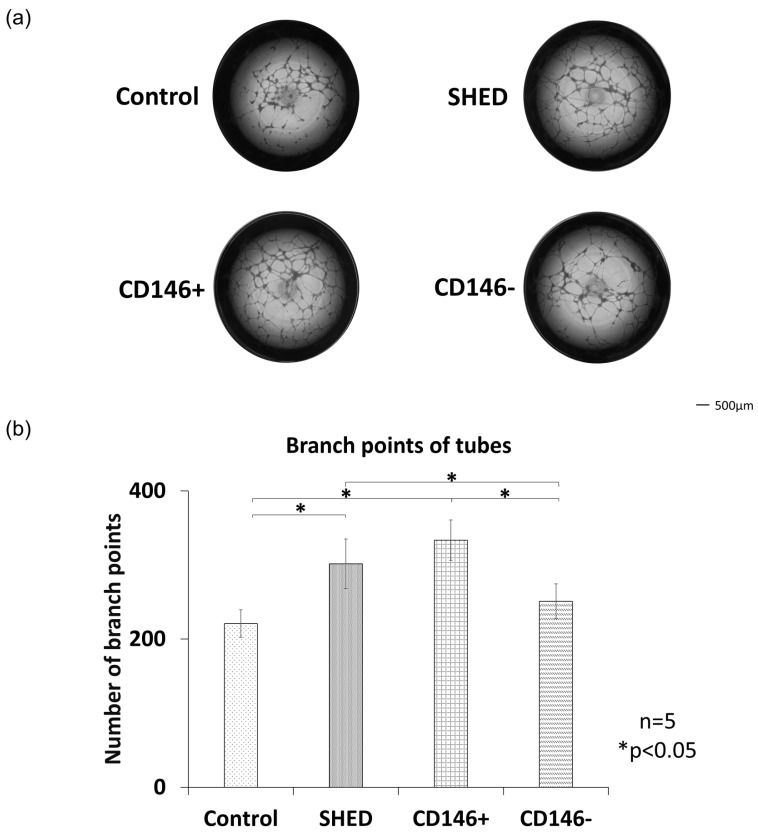
Tube forming assay of human exfoliated deciduous teeth (SHED), CD146 + SHED, and CD146 − SHED. (**a**) Angiogenic potential assessed through co-culture with human umbilical vein endothelial cells (HUVECs). CD146 + SHED exhibited highest number of newly formed vessel branches, followed by SHED, CD146 − SHED, and control group (Scale bar = 500 μm). (**b**) Quantitative analysis revealed that number of vascular branches was highest in CD146 + SHED, with no significant difference observed between CD146 + SHED and SHED. Both CD146 + SHED and SHED demonstrated significantly more vascular branches compared to CD146 − SHED and controls (* *p* < 0.05, Kruskal–Wallis method, *n* = 5).

In the tube formation assay, SHED, CD146 + SHED, and CD146 − SHED were co-cultured with human umbilical vein endothelial cells (HUVECs). Vessel formation was more pronounced in the CD146 + SHED and SHED groups compared to the CD146 − SHED and control groups (Figure 1a). The measurements of vascular bifurcations revealed that bifurcations occurred in the following order: CD146 + SHED > SHED > CD146 − SHED > control. Both CD146 + SHED and SHED showed significantly higher bifurcation counts compared to CD146 − SHED and the control, with no significant differences between CD146 + SHED and SHED (Figure 1b).

### 2.2. Quantitative Reverse Transcription Polymerase Chain Reaction Analysis

**Figure 2:**
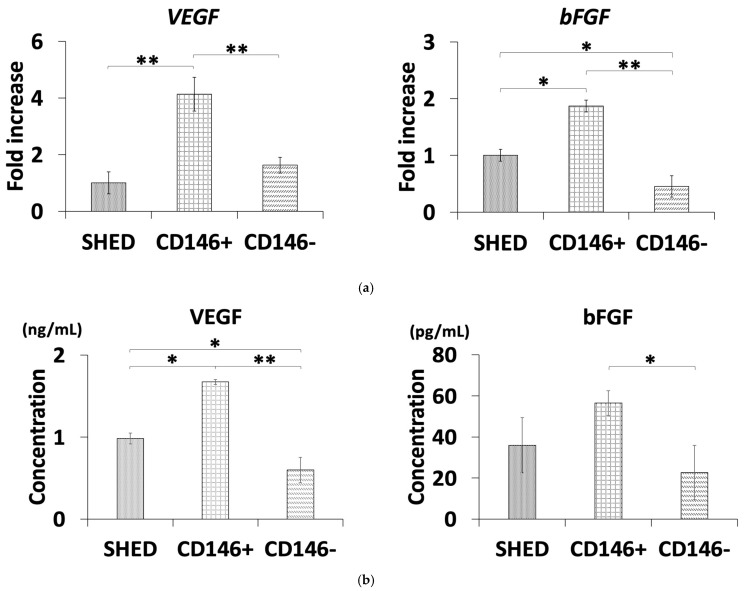
Gene and protein expression levels of angiogenesis-related markers in stem cells from human exfoliated deciduous teeth (SHEDs), CD146 + SHED, and CD146 − SHED. (**a**) Real-time polymerase chain reaction analysis of vascular endothelial growth factor (VEGF) and basic fibroblast growth factor (bFGF) gene expression. VEGF expression levels were significantly higher in CD146 + SHED compared to SHED and CD146 − SHED. Similarly, bFGF expression levels were significantly higher in CD146 + SHED compared to CD146 − SHED, with no significant difference between CD146 + SHED and SHED. (**b**) Enzyme-linked immunosorbent assay (ELISA) was used to analyze VEGF and bFGF protein levels. VEGF protein levels were significantly higher in CD146 + SHED compared to SHED and CD146 − SHED, and SHED also exhibited significantly higher levels compared to CD146 − SHED. bFGF protein levels were significantly higher in CD146 + SHED compared to CD146 − SHED, with trend toward higher levels in CD146 + SHED compared to SHED, although difference was not significant (** *p* < 0.01, * *p* < 0.05, Kruskal–Wallis method, *n* = 5).

CD146 + SHED showed significantly higher levels of VEGF and bFGF gene expression compared to SHED and CD146 − SHED. Moreover, SHED showed significantly higher levels of bFGF gene expression than CD146 − SHED (Figure 2a).

### 2.3. Enzyme-Linked Immunosorbent Assay

CD146 + SHED showed significantly higher protein expression levels of VEGF and bFGF compared to CD146 − SHED. Additionally, SHED showed significantly higher levels of VEGF protein expression than CD146 − SHED (Figure 2a).

## 3. Discussion

MSCs derived from various human tissues exhibit excellent differentiation potential [16], a property used to promote tissue repair by transplanting MSCs into tissue defects. Research on regenerative medicine involving MSCs is being actively conducted worldwide. Within this field, our research has focused on SHEDs. As deciduous teeth naturally exfoliate during permanent tooth replacement, SHED collection is significantly less invasive compared to harvesting BMSCs and DPSCs. Additionally, our previous studies suggested that SHEDs have a bone regenerative potential comparable to that of BMSCs [8,9]. However, SHED collection is limited to a specific period, as deciduous teeth are only available before school age is reached. This time constraint poses a challenge for regenerative therapies involving SHED transplantation. One potential approach involves collecting SHEDs during school-age years for immediate transplantation or preserving SHEDs for future use when transplantation becomes necessary in adulthood. Despite this possibility, long-term storage is associated with the risk of cellular deterioration. Based on these considerations, the ideal use of SHEDs appears to be for transplantation into the cleft jaw of patients with cleft lip and palate. For these patients, autologous iliac bone grafting is typically performed during the canine tooth eruption phase in school-aged children to close the cleft jaw and facilitate canine tooth eruption [9]. Since SHEDs can be easily collected from school-aged children, their use eliminates the need for long-term storage and reduces patient burden by avoiding the harvesting of autologous iliac bone. With this background, research has focused on SHED implantation into the cleft jaw. In a previous study, the bone regenerative potential of SHED, CD146 + SHED, and CD146 − SHED was compared in skull defect models of immunocompromised mice. The results showed that CD146 + SHED exhibited superior bone regenerative capacity [14].

The dental pulp contains numerous blood vessels and houses a specialized microenvironment called the MSC niche [17,18]. This niche consists of MSCs, hematopoietic stem cells, mesenchymal progenitor cells, and vascular pericytes [19]. CD146 is also expressed on the plasma membranes of vascular endothelial cells, pericytes, and MSCs. On MSCs, CD146 serves as a receptor for growth factors such as Netrin-1, Wnt-1, and VEGF [20,21,22]. Additionally, CD146 plays a role in angiogenesis and vascular maintenance by functioning as a co-receptor for VEGFR2 and other factors [20,23,24]. CD146 has been shown to be not only an adhesion molecule but also a cell surface receptor for a variety of ligands, including several growth factors and extracellular matrices. VEGF-A is a major mediator of angiogenesis and binds to the receptor VEGFR2, which is expressed on endothelial cells, macrophages, hematopoietic cells, and smooth muscle cells [13]. Previous studies have found that CD146 interacts with VEGFR2 on endothelial cells and functions as a co-receptor [13]. Furthermore, the inhibition of CD146 using the blocking antibody AA98 or CD146 siRNA suppressed VEGF-A-induced VEGFR2 phosphorylation in HUVECs, demonstrating that the interaction between CD146 and VEGFR2 is essential for functional VEGFR2 signaling [13].

Both MSCs and pericytes are components of the MSC niche and express several common surface antigens, including CD146. Some researchers have proposed that pericytes may be the origin of MSCs, with CD146 + MSCs exhibiting properties closely resembling those of pericytes [25,26,27]. Thus, the hypothesis was formed that CD146 + SHED possess enhanced angiogenic potential, which may explain the observed high bone regeneration capacity.

To compare angiogenic potential, HUVECs were co-cultured with SHED, CD146 + SHED, and CD146 − SHED, and tube formation in HUVECs was evaluated. CD146 + SHED and SHED demonstrated significantly greater vessel formation compared to CD146 − SHED and the control group. Branching point measurements also revealed significantly higher values in CD146 + SHED and SHED compared to CD146 − SHED and the control group.

The gene expression analysis and ELISA results indicated significantly higher levels of VEGF expression in CD146 + SHED compared to SHED and CD146 − SHED. These results supported the enhancement in the HUVEC tube formation co-culture with CD146 + SHED [28,29]. Interestingly, although the co-culture with SHEDs resulted in a comparable number of tube formations as with CD146 + SHED, VEGF expression levels in SHED were significantly lower than those in CD146 + SHED. One possible reason for this is the inhibitory effect of excessive VEGF on HUVEC tube formation. High levels of VEGF can inhibit the migration and proliferation of vascular endothelial cells and pericytes by blocking Notch signaling, thereby suppressing angiogenesis [30,31]. A further examination of the amount of VEGF added to the extracellular matrix (ECM) gel and the number of cells in SHED, CD146 + SHED, and CD146 − SHED is also warranted. Conversely, the genetic analysis and ELISA results for bFGF showed the highest levels in CD146 + SHED, though the difference was not statistically significant compared to SHED. Previous studies have shown that CD146 acts as a co-receptor for VEGFR2, enhancing the expression of bFGF, BMP-2, Runx2, and Osterix through VEGF and VEGFR2 binding [32]. In this study, the observed trend of higher bFGF expression levels in CD146 + SHED may suggest that VEGF-mediated signaling pathways also contribute to bFGF upregulation. Adding growth factors such as VEGF could further promote bFGF expression in CD146 + SHED, warranting further investigation. Angiogenesis is important for osteogenesis, and VEGF in particular plays a role in stimulating the mobilization of blood vessels and osteoclasts and promoting cartilage resorption at the site of repair during the endochondral periosteal ossification phase [2,4]. In this study, the expression levels of angiogenesis-related proteins such as VEGF and bFGF were the highest in CD146 + SHED, which may explain why CD146 + SHED had the highest osteogenic potential in our previous studies. However, the detailed mechanism behind this is a subject for future studies.

This study, along with previous research, suggests that CD146 + SHED has superior angiogenic and bone regeneration ability compared to SHED and CD146 − SHED [14,15]. Furthermore, previous studies have reported that CD146 + MSCs exhibit characteristics closely resembling those of pericytes, CD146 enhances angiogenic effects mediated by VEGF and PDGF, and CD146 functions as a co-receptor for VEGFR2 [13,23,24,32,33]. Thus, CD146 + MSCs further enhance the expression of bFGF, BMP-2, Runx2, and Osterix through VEGF and VEGFR2 signaling. These interactions likely contribute to the observed angiogenesis and bone regeneration. However, the precise mechanisms underlying the enhanced angiogenic potential of CD146 + SHED remain to be explored in future studies.

SHEDs are expected to be used in bone regeneration therapy for the cleft jaw and other areas due to their low invasiveness to patients at the time of harvesting. However, there are some challenges in transplanting SHEDs into the cleft jaw, such as the need to prepare a sufficient number of cells and the scaffold. Although many studies have been published, no gold standard has been established. In fact, previous studies on the clinical application of MSCs implanted in the cleft jaw have varied in terms of cell numbers, scaffolds, growth factors, etc., and the results have not all been favorable [34,35,36,37,38]. In our previous studies, we used an atelocollagen sponge as a scaffold for implanting MSCs into bone defects in mice because of its porous nature and ability to be cultured in three dimensions, which is advantageous for angiogenesis [14]. However, the fabrication of scaffolds using 3D printers is currently under investigation [39]. Three-dimensional printers allow scaffolds to be sculpted to fit the shape of the defective tissue, and the internal structure can be designed as well. The materials used include β-TCP and collagen fibers [39,40]. Optimal scaffolds for angiogenesis and bone regeneration should also be considered for future clinical applications. We would like to investigate scaffolds other than the atelocollagen sponge and use them in animal experiments to evaluate angiogenesis and other processes three-dimensionally.

## 4. Materials and Methods

### 4.1. Cell Isolation and Culture

This study adhered to the Regulations for Epidemiological Research of Hiroshima University Hospital (No. E-20-2) and followed the principles outlined in the Declaration of Helsinki (1964) and subsequent amendments or comparable ethical standards. Informed consent was obtained from all participants. The pulp tissues from extracted deciduous teeth were collected. SHEDs were isolated from the pulp tissue and cultured following the method described in previous studies [14,15,41,42]. To confirm that the isolated SHEDs met the International Society for Cellular Therapy’s definition of MSCs, a preliminary assessment was conducted. MSCs were characterized as cells (1) adhering to plastic containers under standard culture conditions; (2) expressing CD73, CD90, and CD105 while lacking the expression of CD11b or CD14, CD19 or CD79α, CD34, CD45, and HLA-DR; and (3) possessing the capacity to differentiate into osteoblasts, chondrocytes, and adipocytes [43]. These criteria had been verified in earlier research, and SHEDs meeting these conditions were utilized in this study [9]. Therefore, SHEDs were defined as MSCs. Serum-free α-minimal essential medium (α-MEM), supplemented with 0.5 μL/mL penicillin (Meiji Seika Pharma, Tokyo, Japan), 0.24 μL/mL kanamycin (Meiji Seika Pharma), and 1 μL/mL amphotericin (MP Biomedicals, Strasbourg, France), was used for culturing SHEDs.

### 4.2. Fluorescence-Activated Cell Sorting

Pulmonary embolism (PE) Mouse Anti-Human CD146 (BD Pharmingen, San Jose, CA, USA) was added to the suspension of SHEDs (P3-5), and cell sorting for CD146 was performed using FACS Aria II (BD Biosciences, San Jose, CA, USA) and FLOWJO software v10.10 (TOMY Digital Biology, Tokyo, Japan). A κIsotype control (BD Pharmingen) was included as the control, and 7-amino-actinomycin (7-AAD; BD Pharmingen) was used to stain dead cells. CD146-positive cells (CD146 + SHED) and CD146-negative cells (CD146 − SHED) were successfully isolated. The isolated cells were cultured in α-MEM at 37 °C with 5% carbon dioxide (CO_2_). For the experiments described below, comparisons and analyses were conducted among three groups: SHED without cell sorting (SHED), CD146 + SHED, and CD146 − SHED (*n* = 5).

### 4.3. Tube Forming Assay

Endothelial tube formation was assessed using an Endothelial Tube Forming Assay Kit (Cell Biolabs, San Diego, CA, USA). HUVECs (Lonza, Basel, Switzerland) were suspended in EBM-2 (Lonza, Basel, Switzerland) and seeded at a density of 1.0 × 10^5^ cells/well in six-well plates (Corning, NY, USA). Cell culture inserts (Corning, NY, USA) with a pore diameter of 0.4 μm were placed in the wells, with 1.0 × 10^5^ SHED, CD146 + SHED, and CD146 − SHED seeded in the inserts. The co-culture was maintained at 37 °C with 5% CO_2_. The control group included HUVECs that were cultured without co-culture. An ECM gel solution (Cell Biolabs) prepared from mouse sarcoma was added to pre-cooled 96-well plates (Corning, NY, USA) and incubated at 37 °C with 5% CO_2_ for 1 h to solidify the gel. After reaching confluency, 5.0 × 10^4^ HUVECs were collected, suspended in EBM-2, and seeded onto the gel. Cells were incubated at 37 °C with 5% CO_2_ for 12 h. The number of vascular branching points formed in the gel was observed using a BZ-X810 microscope (Keyence, Osaka, Japan) and counted manually.

### 4.4. Quantitative Reverse Transcription Polymerase Chain Reaction Analysis

SHED, CD146 + SHED, and CD146 − SHED cells were cultured at 37 °C with 5% CO_2_ until reaching 80% confluency. Total ribonucleic acid (RNA) was extracted from the collected cells using an RNeasy Mini Kit (Qiagen, Hilden, Germany) and quantified with a NanoDrop One/OneC spectrophotometer (Thermo Fisher Scientific, Inc., Waltham, MA, USA). The reverse transcription of 1 μg total RNA into complementary deoxyribonucleic acid (cDNA) was performed using a ReverTra Ace first-strand cDNA synthesis kit (Toyobo, Osaka, Japan). The expression of VEGF and bFGF was quantified by a quantitative reverse transcription polymerase chain reaction (qRT-PCR) using a Thunderbird SYBR qPCR mix (Toyobo) and a LightCycler^®^ 480 II (Roche Diagnostics, Basel, Switzerland). Relative expression levels were calculated using the ∆∆∆Ct method, normalized to β-actin (ACTB) (Table 1).

### 4.5. Enzyme-Linked Immunosorbent Assay

Cultured cells (P3-5) were collected and centrifuged at 4 °C at 2500 G for 10 min to obtain the cell supernatant. The protein levels of VEGF and bFGF were quantified using ELISA kits (R&D Systems, Minneapolis, MN, USA) according to the manufacturer’s protocol.

### 4.6. Statistical Analysis

Data were presented as the mean ± standard deviation. Differences between the groups were analyzed using the Bonferroni method with the BellCurve for Excel (Social Survey Research Information Co., Ltd, Tokyo, Japan). The results with *p* < 0.05 were considered statistically significant.

## 5. Conclusions

In summary, this study indicated that CD146 + SHED exhibited superior angiogenic potential compared to SHED and CD146 − SHED. Furthermore, CD146 affected the angiogenic potential of SHEDs, highlighting its potential utility in bone regeneration therapies through the implantation of CD146 + SHED. Previous animal-based and cell-based studies have shown that CD146 + SHED has a higher osteogenic and angiogenic potential. However, for clinical application, further studies are needed to elucidate the detailed mechanisms driving angiogenesis and bone regeneration in CD146 + SHED. Specifically, single-cell sorting and clonal culture should be performed in SHED, CD146 + SHED, and CD146 − SHED, and the degree of osteogenic differentiation and the ability to differentiate into angiogenesis in CD146 + SHED and CD146 − SHED populations used in this study should be investigated in detail in future studies. The fabrication and examination of 3D biologic scaffolds using CD146 + SHED and cytokines are needed to achieve optimal bone regeneration in future clinical applications.

## Figures and Tables

**Table 1:** Sequence of each primer.

Gene		Sequence (5′ → 3′)
GAPDH	Forward	CCA CTC CTC CAC CTT TGA
	Reverse	CAC CAC CCT GTT GCT GTA
VEGF	Forward	CCT TGC CTT GCT GCT CTA
	Reverse	CAC CAC TTC GTG ATG ATT CTG
bFGF	Forward	AAC CGT TAC CTG GCT ATG AAG C
	Reverse	TCG TTT CAG TGC CAC ATA CC

## Data Availability

The data that support the findings of this study are available from the corresponding author upon reasonable request.
